# Valor Diagnóstico de Parâmetros Tridimensionais de Strain de Imagem de Speckle Tracking para Detecção de Disfunção Cardíaca Relacionada à Quimioterapia do Câncer: Uma Metanálise

**DOI:** 10.36660/abc.20220370

**Published:** 2023-07-25

**Authors:** Yingying Guan

**Affiliations:** 1 Taizhou Central Hospital Taizhou University Hospital Taizhou China Taizhou Central Hospital (Taizhou University Hospital), Taizhou – China

**Keywords:** Antraciclinas, Cardiotoxicidade, Insuficiência Cardíaca, Tratamento Farmacológico, Neoplasias

## Abstract

**Fundamento:**

Agentes quimioterápicos (por exemplo, antraciclinas, trastuzumabe) comumente usados para o tratamento de tumores malignos demonstraram ter efeitos cardiotóxicos, que estão associados a um prognóstico ruim. A ecocardiografia tridimensional tem sido usada para prever a disfunção cardíaca induzida pela quimioterapia do câncer.

**Objetivos:**

Avaliação do desempenho diagnóstico de parâmetros de strain, área global de strain (AGS), strain longitudinal (SLG), strain circunferencial (SCG) e strain radial (SRG) por metanálise.

**Métodos:**

Estudos relevantes foram pesquisados nas bases de dados Embase, PubMed e Web of Science. A análise estatística foi realizada usando Stata 12. O resumo da curva característica operacional do receptor, sensibilidade, especificidade, razão de verossimilhança positiva (RVP), razão de verossimilhança negativa (RVN), e o correspondente intervalo de confiança de 95% para os quatro parâmetros de strain foram combinados. P<0,05 foi considerado estatisticamente significativo.

**Resultados:**

Nove estudos envolvendo 650 participantes foram incluídos. AGS e SLG mostraram vantagens diagnósticas significativas sobre SCG e SRG. Para AGS, a sensibilidade foi de 0,85 (0,70, 0,93) e a especificidade foi de 0,82 (0,78, 0,86) com RVP de 4,76 (3,55, 6,39) e RVN de 0,18 (0,09, 0,39) e uma área sob a curva (AUC) de 0,85 (0,82, 0,88). Para SLG, a sensibilidade foi de 0,81 (0,74, 0,86) e a especificidade foi de 0,81 (0,68, 0,90) com RVP de 4,35 (2,42, 7,80) e RVN de 0,23 (0,17, 0,33) e uma AUC de 0,85 (0,82, 0,88).OGCS mostrou uma sensibilidade de 0,63 e uma especificidade de 0,79 com uma AUC de 0,77.O SRG mostrou uma sensibilidade de 0,74e uma especificidade de 0,66 com umAUC de 0,73.

**Conclusão:**

Parâmetros 3D-STI de strain AGS e SLG mostraram bom desempenho na detecção precoce de disfunção cardíaca em pacientes com tumores recebendo quimioterapia.

## Introdução

O prognóstico de pacientes com tumores melhorou muito com o desenvolvimento de métodos de terapia tumoral, enquanto as complicações relacionadas aos medicamentos para tratamento de tumores tornaram-se cada vez mais proeminentes, o que tem sido um problema importante que afeta a sobrevida do paciente.^1^ A quimioterapia continua sendo o principal método de tratamento para vários tumores. No entanto, enquanto matam as células cancerígenas, os quimioterápicos também causam danos a muitos tecidos e células normais em todo o corpo, entre os quais o dano às células miocárdicas é uma alteração concomitante comum durante a quimioterapia.^2 - 4^ Por exemplo, foi relatado que as antraciclinas, uma classe de drogas anticancerígenas de amplo espectro altamente eficazes que podem melhorar a sobrevida do paciente e reduzir a recorrência e metástase do tumor, causam cardiotoxicidade relacionada à dose, resultando em dano cardíaco irreversível o que afeta negativamente o prognóstico.^5 , 6^ A incidência geral de cardiotoxicidade (diminuição da fração de ejeção do ventrículo esquerdo [FEVE] > 10% da linha de base e um valor final < 50%) de 9% foi observada em uma coorte heterogênea de 2.625 pacientes com câncer que receberam tratamento contendo antraciclina em uma mediana de 5,2 anos de acompanhamento.^7^ Um estudo anterior relatou uma taxa de disfunção ventricular esquerda de 16%, 32% e 65% (diminuição da fração de ejeção > 10% abaixo do normal) sob dose cumulativas de 300, 400 e 550 mg/m^2^ , respectivamente.^8^ O trastuzumabe, uma terapia direcionada para tumores com superexpressão de HER-2, teve grande sucesso no câncer de mama.^9^ No entanto, foi relatado que o trastuzumabe está associado à perda da função contrátil do VE e à insuficiência cardíaca.^10^ Portanto, a detecção precoce e a prevenção ativa de disfunção cardíaca causada por agentes quimioterápicos contra o câncer são necessários.

A ecocardiografia transtorácica tem sido amplamente recomendada para monitorar a função cardíaca em pacientes recebendo quimioterapia.^11^ Os indicadores tradicionais de FEVE tem várias limitações; particularmente, eles são insensíveis na determinação precoce de mudanças sutis na função miocárdica.^12^ Imagens tridimensionais de *speckle tracking* (3D-STI) é um método de exame ultrassonográfico recém-desenvolvido que pode rastrear a trilha de movimento do speckle acústico do miocárdio na região de interesse no espaço 3D e descrever o grau de deformação do tecido miocárdico para representar a curva de alteração da função cardíaca.^13 , 14^ Comparado com o 2D-STI, o 3D-STI foi mais preciso na identificação dos limites do endocárdio e mostrou superioridade para avaliar o movimento anormal da parede ventricular local e quantificar a massa do VE.^15^ Além disso, o 3D-STI foi mais viável e reprodutível para avaliação da função VE, levando menos tempo do que o 2D-STI.^16^ O 3D-STI é um método não invasivo e altamente eficaz na avaliação da função cardíaca e tem sido aplicado no diagnóstico de disfunção miocárdica subclínica causada por vários patógenos.^17 - 19^ 3D-STI envolve análise multiparâmetro, incluindo área global de *strain* (AGS), *strain* longitudinal (SLG), *strain* radial (SRG), strain circunferencial (SCG). SLG é o parâmetro de *strain* mais bem estudado e demonstrou mostrar valor diagnóstico e prognóstico; as alterações do SLG têm sido consideradas um marcador precoce de cardiotoxicidade.^20^ Mornoş et al. indicaram que o SLG tinha valor preditivo independente para cardiotoxicidade.^21^ Embora os estudos tenham sugerido que o AGS é mais viável do que os parâmetros de *strain* convencionais na detecção precoce da disfunção sistólica do VE, é um parâmetro sensível e repetível.^22 , 23^ Para pacientes com câncer de mama que receberam seis ciclos de quimioterapia com epirrubicina + ciclofosfamida, AGS, SLG e SCG diminuíram acentuadamente durante a quimioterapia intermediária e final, enquanto não houve alterações óbvias no SRG após a quimioterapia; além disso, o AGS mostrou maior área sob a curva (AUC) do que outros parâmetros de *strain* .^24^ No estudo de Galderisi et al., AGS, SLG e SRG mudaram acentuadamente na caracterização de anormalidades iniciais de estrutura e função do VE, enquanto o SCG não mostrou alterações.^25^ Esses estudos mostraram visões inconsistentes sobre o valor clínico desses parâmetros de *strain* na determinação da disfunção cardíaca.

Esta metanálise visa avaliar as vantagens diagnósticas gerais dos quatro parâmetros de *strain* 3D-STI na determinação da lesão da função cardíaca em pacientes com câncer após quimioterapia.

## Métodos

### Recoleção de estudos

DeoEmbase, PubMed e Web of Science, estudos relevantes foram sistematicamente coletados com base na estratégia de busca pré-estabelecida com um tempo de recoleção até 11 de março de 2022. Os termos de busca incluíram quatro categorias: 1) “disfunção” OU “insuficiência cardíaca” OU “cardiotoxicidade” OU “cardiotox”; 2) “quimioterapia” OU “doxorrubicina” OU “daunorrubicina” OU “trastuzumabe” OU “epirubicina” OU “idarubicina” OU “mitoxantrona” OU “antraciclina” OU “ciclofosfamida” OU “Adriamicina” OU “paclitaxel” OU “5-fluorouracil “; 3) “ *three-dimensional speckle tracking* ” OU “ *three-dimensional spot tracking* ” OU ecocardiografia OU ultrassonografia OU ultrassom; e 4) deformação OU *strain* . As quatro categorias de termos de busca foram combinadas com “E”. As estratégias de busca nos diferentes bancos de dado são apresentadas em detalhes na Tabela S1 . Além disso, estudos relevantes na versão em papel também foram coletados manualmente. As referências citadas nos estudos incluídos e revisões relevantes também foram obtidas.

### Seleção de estudo

Os estudos foram incluídos se atendessem aos seguintes critérios: 1) estudos que incluíam pacientes com câncer que receberam quimioterapia pela primeira vez; 2) estudos que registraram o valor diagnóstico de cada parâmetro de *strain* 3D-STI em lesão da função cardíaca, incluindo AGS, SCG, SLG, e SRG; e 3) estudos que forneceram dados em termos de desempenho diagnóstico de cada parâmetro de *strain* de 3D-STI em lesão da função cardíaca, incluindo verdadeiro positivo (VP), verdadeiro negativo (VN), falso positivo (FP), e falso negativo (FN). Estudos que atenderam aos seguintes critérios foram excluídos desta metanálise: 1) resumos de conferências, comentários, revisões, e outros artigos não originais; 2) estudos que incluíram pacientes com histórico de cardiopatia ou pacientes que receberam previamente medicamentos ou radioterapia que causam cardiotoxicidade; e 3) estudos múltiplos que relataram os dados das mesmas populações participantes, apenas o estudo com os dados mais completos para análise foi incluído.

### Extração de dados e avaliação de qualidade

Os dados foram extraídos dos estudos incluídos por dois investigadores independentes (YY-Guan e JY-Zhou), e qualquer diferença foi resolvida por meio de consulta. Os dados extraídos incluíram o seguinte: ano de publicação, nome do primeiro autor, região da pesquisa e informações sobre os participantesenvolvidonos estudos, incluindo tamanho da amostra, idade, sexo, VP, FP, VN, FN, tipos de câncer e esquemas quimioterápicos. A definição de disfunção cardíaca e os fornecedores do sistema de ultra-som também foram extraídos. QUADAS 2^26^ foi usado para avaliar a qualidade metodológica dos estudos.

### Análise estatística

A análise estatística foi realizada usando o comando MIDAS (modelo de efeito misto bivariado) fornecido no Stata 12 (versão 12 SE). A sensibilidade, especificidade, razões de verossimilhança positiva e negativa (RVP e RVN, respectivamente), curva resumida da característica operacional do receptor (SROC), e correspondente intervalo de confiança (IC) de 95% foram relatados. O teste I^2^ e o teste Q de Cochran foram usados para avaliar a heterogeneidade dos estudos,^27^ e a heterogeneidade significativa foi detectada quando o valor-p da estatística Q foi < 0,05 e/ou I^2^ > 50%. A meta-regressão foi conduzida para investigar as influências das regiões, a definição de disfunção cardíaca, fornecedores e o tipo de câncer nos resultados agrupados. O viés de publicação entre os estudos foi avaliado usando o gráfico de funil de Deek.^28^ A análise de correlação de Spearman foi usada para avaliar os efeitos do limiar. Um valor de p < 0,05 indicou a presença de um efeito de limiar significativo.^29^

## Resultados

### Recoleção de estudos

**Figura 1:**
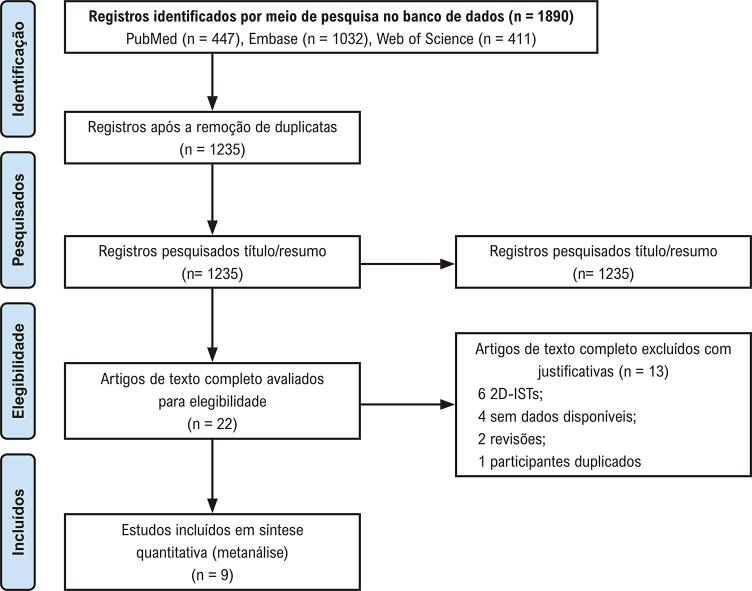
– Fluxograma do processo de seleção dos estudos.

Foram coletados dos bancos de dados Embase, PubMed e Web of Science, 1.032, 447 e 411 estudos, respectivamente. Destes, 655 estudos duplicados foram excluídos; 1213 estudos irrelevantes foram removidos pela leitura do título/resumo. Entre os 22 estudos restantes, seis estudos envolvendo 2D-STIs, quatro sem dados disponíveis, duas revisões, e um estudo envolvendo participantes duplicados foram excluídos por leitura do texto completo. Como resultado, nove estudos.^21 , 24 , 30 - 36^ que atenderam aos critérios de inclusão e exclusão pré-definidos foram selecionados para esta metanálise ( Figura 1 ).

### Características dos estudos incluídos

Todos os nove estudos incluídos eram estudos de coorte prospectivos publicados entre 2014 e 2021, e um total de 650 participantes foram incluídos, com 185 homens e 465 mulheres. A Tabela 1 apresenta informações detalhadas sobre os nove estudos incluídos. Esses estudos foram conduzidos na China, Portugal, Romênia, e Grécia.Entre os nove estudos incluídos, pacientes com câncer de mama foram analisados em três estudos,^24 , 30 , 31^ pacientes com linfoma não Hodgkin difuso de grandes células B foram examinados em dois estudos^33 , 34^ e pacientes com câncer colorretal,^35^ linfoma não-Hodgkin^32^ e câncer de ovário^36^ foram incluídos em um estudo. Em um estudo de Mornoş et al.,^21^ vários tipos de câncer estavam envolvidos, incluindo leucemia linfoblástica aguda, leucemia mieloblástica aguda, câncer de mama, linfoma não-Hodgkin, linfoma de Hodgkin e osteossarcoma. Os pacientes foram confirmados histopatologicamente ou citopatologicamente em quatro estudos,^24 , 31 , 33 , 34^ e nenhum dado disponível foi relatado nos outros cinco estudos.^21 , 30 , 32 , 35 , 36^ Uma diminuição na FEVE foi usada como critério para determinar a função cardíaca prejudicada em cinco estudos,^21 , 30 - 32 , 34^ e a alegação de que a cardiotoxicidade ocorreu em pacientes com câncer após a quimioterapia foi sugerida nos quatro estudos restantes.^24 , 33 , 35 , 36^ Além do sistema de ultrassom Philips utilizado no estudo de Song et al.^33^ e Wang et al.,^34^ um sistema de ultrassom Doppler colorido Vivid E9 (GE Healthcare) foi usado nos outros estudos, conforme mostrado na Tabela S2. O valor de cortes de VP, FP, VN, e FN para AGS, SLG, SCG, e parâmetros SRG de 3D-STI no diagnóstico de lesão da função cardíaca são apresentados na Tabela 2 . A avaliação da qualidade metodológica mostrou que o viés desses estudos foi moderado, indicando uma qualidade metodológica moderada ( Figura S1 ).

**Tabela 1:** – Características de 9 estudos incluídos nesta metanálise

Estudo	Área	Tipo de câncer	Confirmado de câncer	Regime de quimioterapia	n, M/F	Idade, anos	DC (%)	Definição de DC
Chen, J 2019	China	Câncer de mama	Patologicamente	EC	83, 0/83	49,25±8,75	25,0%	DSVE após receber quimioterapia
Coutinho Cruz, M 2020	Portugal	Câncer de mama	NR	Antraciclinas	105, 0/105	53,8±12,5	21,0%	Redução absoluta da FEVE > 10% para um valor < 54%
Guan, J 2021	China	Câncer de mama	Histologia ou citopatologia	G1: T+Cb, G2: A+E+C+T, G3: H+G2	79, 0/79	48 (22-66)	11,4%	Redução da FEVE em ≥5% a <53% com sintomas de insuficiência cardíaca ou uma redução assintomática da FEVE em ≥10% a <53%
Mihalcea, D 2020	Romênia	NHL	NR	CHOP	110, 51/59	58±11	15,6%	Redução da FEVE abaixo de 50%, com mais de 10 pontos percentuais
Mornos, C 2014	Grécia	Mama, NHL, HL, ALL, AML, Osteossarcoma	NR	Antraciclinas	59, 24/35	51±10	13,6%	Redução da FEVE em ≥5% a <55% com sintomas de insuficiência cardíacaou uma redução assintomática da FEVE em ≥10% a <55%
Song, FY 2017	China	DLBCL	Histopatologicamente	R-CHOP	89, 60/29	49,3±12,5	50,0%	Disfunção sistólica subclínica após receber quimioterapia
Wang, B 2020	China	DLBCL	Histopatologicamente	(R)-CHOP	65, 31/34	51,3±13,5	12,3%	Redução da FEVE superior a 10%, para um valor <53%, confirmada por ecocardiograma repetido
Wang, Z 2021	China	CRC	NR	mFOLFOX6	30, 19/11	50,2±7,45	75,0%	A toxicidade latente no miocárdio ventricular esquerdo após receber quimioterapia
Zhai, Z 2021	China	Cáncer do ovário	NR	Carboplatina+ Paclitaxel	30, 0/30	51,6±7,4	50,0%	DSVE após receber quimioterapia

*ALL, Leucemia linfoblástica aguda; AML, Leucemia mieloblástica aguda; HL, linfoma de Hodgkin; T, Docetaxel; Cb, Carboplatina; A, Doxorrubicina; E, Epirrubicina; C, Ciclofosfamida; H, Trastuzumabe; NHL, linfoma não-Hodgkin; CRC, câncer colorretal; EC, ciclofosfamida combinada com epirrubicina; (R)-CHOP, (rituximabe)+ciclofosfamida+doxorrubicina+vincristina+prednisona; mFOLFOX6, oxaliplatina+5-fluorouracil+leucovorina cálcica; DLBCL, linfoma não Hodgkin difuso de grandes células B; F, feminino; M, masculino; NR, não informado; DC, disfunção cardíaca; DSVE, disfunção sistólica ventricular esquerda; FEVE, fração de ejeção do ventrículo esquerdo. P<0,05 foi considerado estatisticamente significativo em todos os estudos incluídos.*

**Tabela 2:** – TP/FN/FP/TN dos parâmetros 3D-STI

Estudo	País	Corte	Verdadeiro positivo	Falso negativo	Falso positivo	Verdadeiro negativo
**AGS**						
Chen, J 2019	China	-31,5%	68	15	49	200
Mihalcea, D 2020	Romênia	-28,0%	15	2	16	76
Wang, Z 2021	China	-28,0%	64	26	4	26
Zhai, Z 2021	China	-32,0%	29	1	3	26
**SLG**						
Chen, J 2019	China	-16,5%	62	21	116	133
Guan, J 2021	China	-22,7%	5	4	4	66
2Mihalcea, D 2020	Romênia	-19,0%	16	2	14	78
Mornos, C 2014	Grécia	-13,7%	7	1	15	36
Song, FY 2017	China	-20,4%	71	18	27	62
Wang, B 2020	China	-13,8%	6	2	5	49
Wang, Z 2021	China	-20,0%	80	10	11	19
Zhai, Z 2021	China	-17,0%	25	5	1	29
**SCG**						
Chen, J 2019	China	-17,5%	57	26	116	133
Coutinho Cruz, M 2020	Portugal	-34,2%	14	8	20	55
Guan, J 2021	China	-16,5%	6	3	13	57
Mihalcea, D 2020	Romênia	-37,0%	14	4	17	74
Song, FY 2017	China	-29,2%	62	27	36	53
Wang, Z 2021	China	-18,0%	19	71	1	29
Zhai, Z 2021	China	-19,0%	25	5	3	27
**SRG**						
Chen, J 2019	China	44,5%	50	33	103	146
Coutinho Cruz, M 2020	Portugal	34,4%	16	6	25	55
Mihalcea, D 2020	Romênia	43,0%	11	7	28	64
Wang, Z 2021	China	45,0%	67	23	15	15
Zhai, Z 2021	China	50,0%	27	3	7	23

*AGS: área global de strain; SLG: strain longitudinal; SCG: strain circunferencial; SRG: strain radial.*

### Valor diagnóstico da AGS

**Figura 2:**
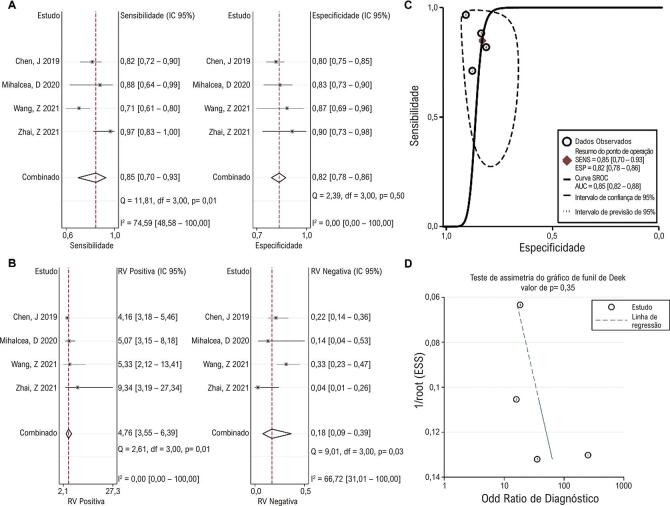
– Resultados agrupados mostrando o valor de diagnóstico de AGS. Gráfico de metanálise mostrando o pool sensibilidade e especificidade (A) e RVP e RVN (B) de AGS no diagnóstico de lesão da função cardíaca em pacientes em tratamento quimioterápico. As curvas SROC mostram o desempenho diagnóstico do AGS (C). O teste de assimetria do gráfico de funil de Deek mostra viés de publicação entre os estudos (D). RVP: razão de verossimilhança positiva; RVN: razão de verossimilhança negativa; SROC: resumo das características operacionais do receptor; AGS: área global de strain.

O valor diagnóstico da AGS em 3D-STI para lesão da função cardíaca em pacientes com câncer foi relatado em quatro estudos. A análise de correlação de Spearman não revelou efeito limiar significativo (p = 1,00). A sensibilidade combinada foi de 0,85 (0,70, 0,93) com heterogeneidade significativa. A especificidade combinada foi de 0,82 (0,78, 0,86) e nenhuma heterogeneidade significativa foi observada ( Figura 2A ). A RVP agrupada foi de 4,76 (3,55, 6,39) sem heterogeneidade significativa, enquanto a RVN agrupada foi de 0,18 (0,09, 0,39) com heterogeneidade significativa ( Figura 2B ).A curva SROC mostrou uma área sob a curva (AUC) de 0,85 (0,82, 0,88), sugerindo o bom desempenho diagnóstico da AGS em predizer lesão da função cardíaca em pacientes submetidos à quimioterapia ( Figura 2C ). Não houve viés de publicação significativo entre os quatro estudos ( Figura 2D ).

### Valor de diagnóstico de SLG

**Figura 3:**
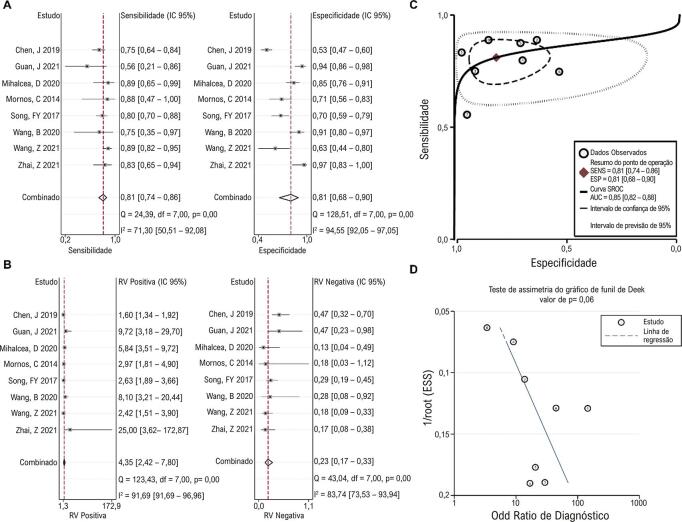
– Resultados agrupados mostrando ovalor de diagnóstico de SLG. Gráfico de metanálise mostrando o pool sensibilidade e especificidade (A) e RVP e RVN (B) de AGS no diagnóstico de lesão da função cardíaca em pacientes em tratamento quimioterápico. As curvas SROC mostram o desempenho diagnóstico do AGS (C). O teste de assimetria do gráfico de funil de Deek mostra viés de publicação entre os estudos (D). RVP: razão de verossimilhança positiva; RVN: razão de verossimilhança negativa; SROC: resumo das características operacionais do receptor; SLG: strain longitudinal global.

O valor diagnóstico de SLG em 3D-STI para lesão da função cardíaca em pacientes com câncer foi relatado em oito estudos. A análise de correlação de Spearman não detectou efeito limiar significativo (p = 0,05). A sensibilidade e especificidade combinadas foram 0,81 (0,74, 0,86) e 0,81 (0,68, 0,90), e heterogeneidade significativa foi encontrada entre esses estudos ( Figura 3A ). As RVP e RVN combinadas foram 4,35 (2,42, 7,80) e 0,23 (0,17, 0,33), respectivamente, e foi observada heterogeneidade significativa entre os estudos ( Figura 3B ). A curva SROC mostrou uma AUC de 0,85 (0,82, 0,88), sugerindo bom desempenho diagnóstico do SLG em predizer lesão da função cardíaca em pacientes submetidos à quimioterapia ( Figura 3C ). Nenhum viés de publicação significativo foi observado ( Figura 3D ).

### Valor de diagnóstico do SCG

**Figura 4:**
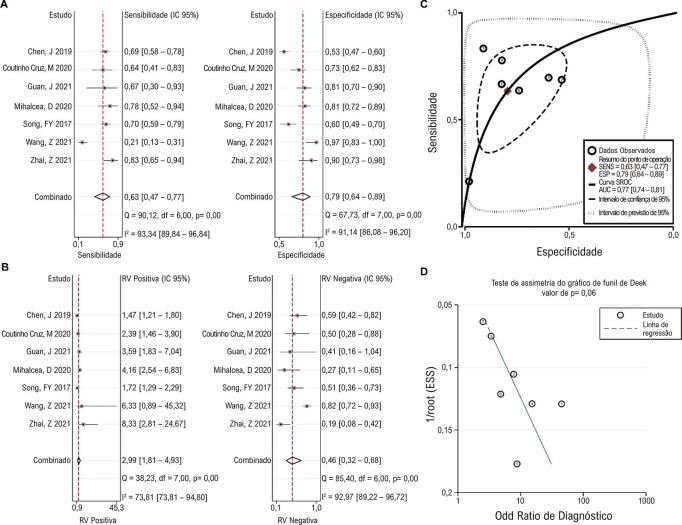
– Resultados agrupados mostrando ovalor diagnóstico de SCG. Gráfico de metanálise mostrando o pool sensibilidade e especificidade (A) e RVP e RVN (B) de AGS no diagnóstico de lesão da função cardíaca em pacientes em tratamento quimioterápico. As curvas SROC mostram o desempenho diagnóstico do AGS (C). O teste de assimetria do gráfico de funil de Deek mostra viés de publicação entre os estudos (D). RVP: razão de verossimilhança positiva; RVN: razão de verossimilhança negativa; SROC: resumo das características operacionais do receptor; SCG: strain circunferencial global.

Sete estudos relataram o valor diagnóstico da SCG em 3D-STI para lesão da função cardíaca em pacientes com câncer. A análise de correlação de Spearman não encontrou nenhum efeito de limiar significativo (p = 0,39). Foi observada heterogeneidade significativa entre os estudos em termos de sensibilidade e especificidade. A sensibilidade e especificidade combinadas foram 0,63 (0,47, 0,77) e 0,79 (0,64, 0,89), respectivamente ( Figura 4A ). As RVP e RVN combinadas foram 2,99 (1,81, 4,93) e 0,46 (0,32, 0,68), respectivamente, e foi observada heterogeneidade significativa entre os estudos ( Figura 4B ). A curva SROC indicou uma AUC de 0,77 (0,74, 0,81) ( Figura 4C ). Nenhum viés de publicação significativo entre os estudos foi detectado usando o gráfico de funil de Deek ( Figura 4D ).

### Valor de diagnóstico de SRG

**Figura 5:**
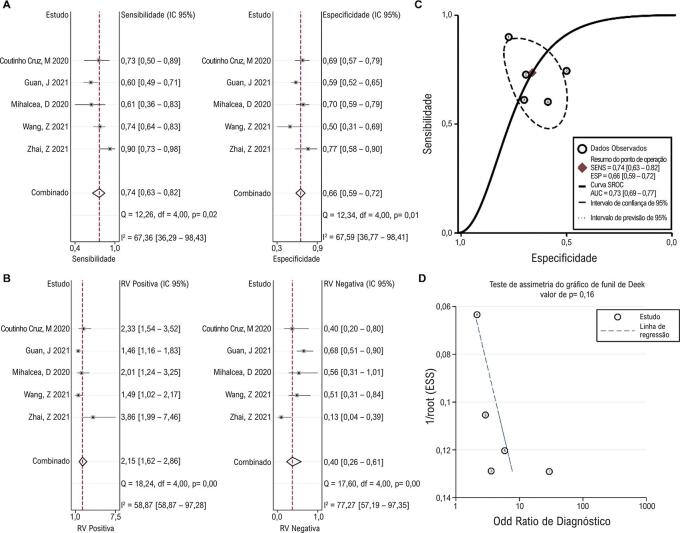
– Resultados agrupados mostrando ovalor diagnóstico de SRG. Gráfico de metanálise mostrando o pool sensibilidade e especificidade (A) e RVP e RVN (B) de AGS no diagnóstico de lesão da função cardíaca em pacientes em tratamento quimioterápico. As curvas SROC mostram o desempenho diagnóstico do AGS (C). O teste de assimetria do gráfico de funil de Deek mostra viés de publicação entre os estudos (D). RVP: razão de verossimilhança positiva; RVN: razão de verossimilhança negativa; SROC: resumo das características operacionais do receptor; SRG: strain radial global.

Cinco estudos relataram o valor diagnóstico do SRG em 3D-STI para lesão da função cardíaca em pacientes com câncer. A análise de correlação de Spearman não indicou nenhum efeito de limiar significativo (p = 1,00). A sensibilidade e especificidade agrupadas foram 0,74 (0,63, 0,82) e 0,66 (0,59, 0,72), respectivamente, e foi observada heterogeneidade significativa entre os estudos ( Figura 5A ). Uma heterogeneidade significativa para RVP e RVN foi observada entre os estudos. Os valores combinados de RVP e RVN foram 2,15 (1,62, 2,86) e 0,40 (0,26, 0,61), respectivamente ( Figura 5B ). A curva SROC mostrou uma AUC de 0,73 (0,69, 0,77) ( Figura 5C ). Não foi observado viés de publicação significativo entre os estudos ( Figura 5D ).

### Meta-regressão

A meta-regressão foi realizada para investigar as influências das regiões, a definição de disfunção cardíaca, fornecedores e o tipo de câncer na sensibilidade e especificidade agrupadas ( Tabela 3 ). A meta-regressão não foi realizada para AGS porque apenas quatro estudos relataram o valor diagnóstico de AGS para prever disfunção cardíaca. Para SCG, todas essas variáveis não tiveram influência significativa na sensibilidade e especificidade agrupadas. Para SLG, a definição de disfunção cardíaca e tipo de câncer influenciou significativamente a sensibilidade agrupada. Para SRG, as regiões mostraram influências significativas na especificidade agrupada, e as influências do tipo de câncer na sensibilidade e especificidade agrupadas foram estatisticamente significativas.

**Tabela 3:** – Resultados da meta-regressão

Fatores	Categoria	Nº de estudos	Sensibilidade	Valor p	Especificidade	Valor p
**AGS**						
Regiões	China	6	0,80 (0,73, 0,87)	0,1	0,82 (0,70, 0,94)	0,93
	Europa	2	0,89 (0,76, 1,00)		0,79 (0,56, 1,00)	
Definição de DC	Redução da FEVE	4	0,79 (0,66, 0,92)	0,04	0,87 (0,78, 0,96)	0,77
	Toxicidade da quimioterapia	4	0,82 (0,76, 0,88)		0,72 (0,56, 0,89)	
Fornecedores	GE	6	0,82 (0,75, 0,89)	0,1	0,81 (0,68, 0,94)	0,68
	Philips	2	0,79 (0,68, 0,90)		0,82 (0,62, 1,00)	
Tipo de câncer	Mama	2	0,69 (0,55, 0,83)	<0,01	0,80 (0,58, 1,00)	0,64
	Não mama	6	0,83 (0,78, 0,89)		0,82 (0,69, 0,94)	
**SLG**						
Regiões	China	5	0,60 (0,42, 0,79)	0,45	0,79 (0,64, 0,94)	0,78
	Europa	2	0,71 (0,44, 0,98)		0,78 (0,56, 1,00)	
Definição de DC	Redução da FEVE	3	0,70 (0,47, 0,93)	0,77	0,79 (0,62, 0,96)	0,69
	Toxicidade da quimioterapia	4	0,60 (0,40, 0,79)		0,78 (0,61, 0,95)	
Fornecedores	GE	6	0,62 (0,45, 0,80)	0,69	0,81 (0,70, 0,92)	0,31
	Philips	1	0,70 (0,35, 1,00)		0,60 (0,21, 0,98)	
Tipo de câncer	Mama	3	0,65 (0,41, 0,89)	0,96	0,70 (0,50, 0,90)	0,11
	Não mama	4	0,61 (0,41, 0,82)		0,84 (0,72, 0,97)	
**SCG**						
Regiões	China	3	0,75 (0,62, 0,87)	0,83	0,62 (0,54, 0,70)	0,01
	Europa	2	0,68 (0,48, 0,88)		0,69 (0,61, 0,77)	
Definição de DC	Redução da FEVE	2	0,68 (0,48, 0,88)	0,25	0,69 (0,61, 0,77)	0,28
	Toxicidade da quimioterapia	3	0,75 (0,62, 0,87)		0,62 (0,54, 0,70)	
Tipo de câncer	Mama	2	0,65 (0,53, 0,77)	0,01	0,63 (0,54, 0,71)	0,04
	Não mama	3	0,77 (0,69, 0,86)		0,67 (0,58, 0,76)	

*AGS: área global de strain; SLG: strain longitudinal; SCG: strain circunferencial; SRG: strain radial; DC: disfunção cardíaca; FEVE: fração de ejeção do ventrículo esquerdo.*

## Discussão

### Principais Descobertas

Esta metanálise revelou que os parâmetros de *strain* 3D-STI AGS e SLG mostraram alta sensibilidade (0,85 para AGS, 0,81 para SLG) e especificidade (0,82 para AGS, 0,81 para SLG) no diagnóstico de lesão da função cardíaca em pacientes com câncer após quimioterapia, com AUC de 0,85 para ambos parâmetros de *strain* , sugerindo bom desempenho diagnóstico. SCG e SRG mostraram vantagens diagnósticas relativamente pobres para determinar a lesão da função cardíaca em pacientes com câncer após quimioterapia, com uma sensibilidade de 0,63 e uma especificidade de 0,79 com uma AUC de 0,77 para SCG e uma sensibilidade de 0,74 e uma especificidade de 0,66 com uma AUC de 0,73 para SRG ( Figura Central ).

**Figura Central:**
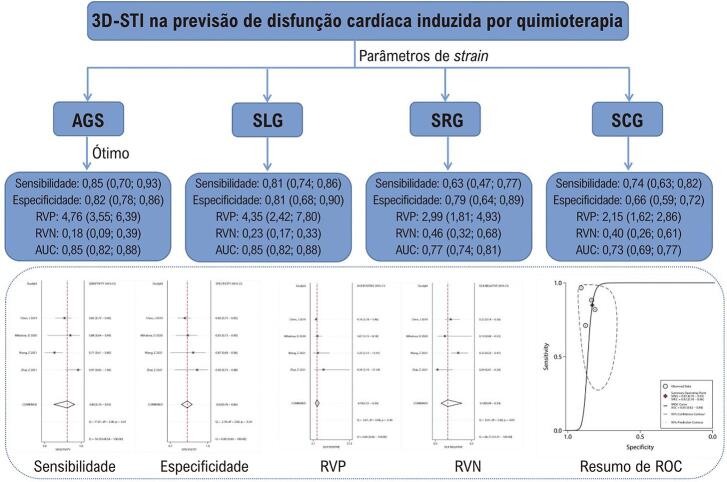
: Valor Diagnóstico de Parâmetros Tridimensionais de Strain de Imagem de Speckle Tracking para Detecção de Disfunção Cardíaca Relacionada à Quimioterapia do Câncer: Uma Metanálise

*Figura central dos resultados do artigo. Quatro parâmetros 3D-STI de strain na previsão de disfunção cardíaca relacionada à quimioterapia do câncer foram avaliados por metanálise. O AGS apresentou o melhor desempenho diagnóstico.*

### Desempenho de diagnóstico de cada parâmetro em estudos anteriores

Estudos anteriores demonstraram que o AGS é o parâmetro mais sensível de *strain* refletindo alterações da função miocárdica ventricular esquerda.^23 , 37^ Para pacientes com fatores de risco para o desenvolvimento de insuficiência cardíaca, o AGS apresentou sensibilidade de 86,3% e especificidade de 88,4% na determinação de disfunção sistólica ventricular esquerda precoce.^23^ Piveta et al. indicaram que, em pacientes com câncer de mama que receberam uma dose cumulativa mais baixa de doxorrubicina (120 mg/m^2^ ), apenas o parâmetro 3D-STI de strain AGS mudou, e sua mudança foi correlacionada com diminuição da FEVE (cardiotoxicidade definitiva).^38^ A AGS é um índice introduzido tardiamente, representando o percentual de *strain* na superfície endocárdica do VE. As vantagens diagnósticas da AGS são provavelmente alcançadas por uma avaliação mais abrangente, integrando ambos movimentos, o longitudinal e o circunferencial do miocárdio.^23^ Além disso, o endocárdio é o mais propenso à isquemia, o que explica razoavelmente o bom desempenho da AGS em detectar alterações na função ventricular esquerda.^23^ Oikonomou et al. indicaram que o SLG teve um bom desempenho preditivo para disfunção cardíaca relacionada à terapia do câncer com uma odd ratio de 12,27 e AUC de 0,86.^39^ A redução do SLG foi preditiva de diminuição da FEVE em pacientes recebendo antraciclinas. Para pacientes com tumores com FEVE normal, um SLG comprometido ≥ -18% mostrou bom valor preditivo para aumento do risco de disfunção cardíaca induzida por antraciclina (definido como uma diminuição na fração de ejeção > 10% e valor final < 50%) e aumento da mortalidade cardiovascular.^40^ Para pacientes com tumores recebendo uma dose cumulativa de antraciclina de 150 mg/m2, o SLG obtido (limiar de -17,45%) mostrou valor preditivo mais forte para cardiotoxicidade, com uma AUC de 0,82, e pode ser usado como um fator preditivo independente.^41^ Durante a avaliação da variação funcional da sístole segmentar do ventrículo esquerdo para pacientes com linfoma recebendo quimioterapia com antraciclina, uma redução significativa no 3D-SLG da linha de base foi observada após quatro ciclos de tratamento com antraciclina, enquanto nenhuma variação na 3D-SCG foi observada.^19^ Chen et al. avaliaram o dano miocárdico precoce em pacientes com câncer de mama recebendo quimioterapia com antraciclina usando 3D-STI, e nenhuma variação no SRG foi observada após a terapia com antraciclina.^24^ Para avaliar a variação funcional no miocárdio do ventrículo direito em pacientes com câncer de mama usando 3D-STI, AGS e SLG do ventrículo direito diminuíram acentuadamente, enquanto SCG e SRG permaneceram inalterados após quimioterapia com pirarubicina.^42^ Consistentemente com os resultados de nossa metanálise, os achados desses estudos sugeriram que AGS e SLG tinham bom valor preditivo, enquanto SCG e SRG mostraram vantagens diagnósticas baixas na detecção de cardiotoxicidade em pacientes com tumores recebendo quimioterapia. No entanto, a superioridade no desempenho diagnóstico do AGS é obtida com base em apenas quatro estudos e deve ser mais confirmada.

### Causas de heterogeneidade

Houve heterogeneidade significativa entre os estudos em relatar alguns índices de resultados, e também houve diferenças nos critérios de diagnóstico, regime de quimioterapia, tipo de câncer, e critérios para comprometimento da função cardíaca entre os participantes em diferentes estudos. A meta-regressão foi realizada para investigar as fontes potenciais de heterogeneidade e descobriu que a definição de disfunção cardíaca (redução da FEVE e toxicidade da quimioterapia), regiões que o estudo conduziu (China e Europa) e tipo de câncer (câncer de mama e câncer não-mama) podem ser fontes de heterogeneidade, especialmente o tipo de câncer. O tipo de câncer mostrou influências significativas na sensibilidade agrupada do SLG e na sensibilidade e especificidade do SRG. Os parâmetros de *strain* 3D mostraram intermediários de concordância pobre, e foi sugerido usar a mesma plataforma 3D STE para obter os dados iniciais e de acompanhamento em estudos longitudinais.^43^ Entre os estudos incluídos nesta metanálise, o sistema de ultrassom VividE9 foi usado na maioria dos estudos, enquanto o sistema de ultrassom Philips também foi usado em dois estudos. Na análise de meta-regressão, os fornecedores não mostraram influências significativas na sensibilidade (p=0,1 para SLG e p=0,69 para SCG) e especificidade (p=0,68 para SLG e p=0,31 para SCG), indicando que o fornecedor não era uma fonte de heterogeneidade.

### Pontos fortes e limitações

O desempenho de diagnóstico dos quatro parâmetros de *strain* 3D-STI na detecção de disfunção cardíaca em pacientes com tumores recebendo quimioterapia foi amplamente avaliado nesta metanálise. Dentre estes, o AGS e o SLG apresentaram boas perspectivas de aplicação neste campo. A qualidade metodológica dos estudos na metanálise atual foi moderada e nenhum viés de publicação significativo foi detectado nesses estudos, indicando a alta confiabilidade dos resultados. No entanto, este estudo tem algumas limitações. Primeiro, a meta-regressão não foi realizada para AGS porque apenas quatro estudos relataram o valor diagnóstico da AGS para prever disfunção cardíaca. Em segundo lugar, embora a AUC para SLG pareça boa, os valores de corte variaram muito entre os estudos (de -13,7% a -22,7%). Isso foi comum em metanálises de ensaios diagnósticos, provavelmente atribuído às diferenças nos critérios diagnósticos, número de participantes, esquema quimioterápico, tipo de câncer e critérios para comprometimento da função cardíaca entre os participantes em diferentes estudos. Além disso, os resultados da meta-regressão indicaram que o tipo de câncer influenciou significativamente a sensibilidade agrupada do SLG. Em terceiro lugar, o número de estudos incluídos e participantes envolvidos foi pequeno, e mais estudos de alta qualidade com amostras grandes são necessários para verificar a estabilidade e extrapolação dos resultados.

## Conclusão

Em conclusão, esta metanálise indicou que os parâmetros de *strain* 3D-STI AGS e SLG mostraram bom desempenho na detecção de disfunção cardíaca em pacientes com tumores recebendo quimioterapia. No entanto, a superioridade no desempenho diagnóstico do AGS é obtida com base em apenas quatro estudos. Portanto, mais investigações são necessárias para confirmar esses achados com base em estudos de grande amostra de alta qualidade. Clinicamente, além do SLG, mais atenção deve ser dada às alterações do AGS ao avaliar com 3D-STI a disfunção cardíaca em pacientes com tumores recebendo quimioterapia.

## *Material suplementar

Para tabela suplementar 1, por favor, clique aqui



Para tabela suplementar 2, por favor, clique aqui



Para figura suplementar, por favor, clique aqui


