# Supplementary material for: Valor Diagnóstico de Parâmetros Tridimensionais de Strain de Imagem de Speckle Tracking para Detecção de Disfunção Cardíaca Relacionada à Quimioterapia do Câncer: Uma Metanálise
Source: Arq Bras Cardiol. 2023 Jul 25;120(8):e20220370. [Article in Portuguese] doi: 10.36660/abc.20220370 (PMC10464855; doi:10.36660/abc.20220370)
Supplement: Supplementary file 1 [file 2022-0370_AO_SupplementaryTable1.pdf]

**Table S1. Detailed search strategies for different databases**

The detailed retrieval strategy from PubMed database

| Search | Query | Items found |
|---|---|---|
| #1 | (cardiotoxicity[mh] OR cardiotox*[tw] OR “heart failure”[mh] OR “heart failure”[tw] OR dysfunction[tw]) | 731221 |
| #2 | (chemotherapy[mh] OR chemotherap*[tw] OR trastuzumab[tw] OR anthracycline[tw] OR doxorubicin[tw] OR adriamycin[tw] OR idarubicin[tw] OR epirubicin[tw] OR daunorubicin[tw] OR mitoxantrone[tw] OR 5-fluorouracil[tw] OR paclitaxel[tw] OR cyclophosphamide[tw]) | 1856239 |
| #3 | ((three-dimensional speckle tracking[tiab]) OR (three-dimensional spot tracking[tiab]) OR echocardiography[mh] OR echocardiogr*[tw] OR ultrasonography[tw] OR ultrasound[tw]) | 688789 |
| #4 | (deformation[tw] OR strain[tw]) | 521080 |
| #5 | #1 AND #2 AND #3 AND #4 | 447 |

The detailed retrieval strategy from Embase database

| Search | Query | Items found |
|---|---|---|
| #1 | cardiotoxicity.mp. or exp cardiotoxicity/ | 52736 |
| #2 | (cardiotox* or "heart failure" or dysfunction).ab,kw,ti. | 965208 |
| #3 | #1 OR #2 | 993570 |
| #4 | chemotherapy.mp. or exp chemotherapy/ | 1001185 |
| #5 | (chemotherapy or trastuzumab or anthracycline or doxorubicin or adriamycin or idarubicin or epirubicin or daunorubicin or mitoxantrone or 5-fluorouracil or paclitaxel or cyclophosphamide).ab,kw,ti | 827805 |
| #6 | #4 OR #5 | 1149434 |
| #7 | echocardiography.mp. or exp echocardiography/ | 408233 |
| #8 | ("three-dimensional speckle tracking" or "three-dimensional spot tracking" or echocardiography or ultrasonography or ultrasound).ab,kw,ti | 742103 |
| #9 | #7 OR #8 | 931694 |
| #10 | (deformation or strain).ab,kw,ti | 561928 |
| #11 | #3 AND #6 AND #9 AND #10 | 1032 |

The detailed retrieval strategy from Web of Science database

| Search | Query | Items found |
|---|---|---|
| #1 | (cardiotoxicity OR cardiotox* OR “heart failure” OR dysfunction) (All Fields) | 820886 |
| #2 | (chemotherapy OR trastuzumab OR anthracycline OR doxorubicin OR adriamycin OR idarubicin OR epirubicin OR daunorubicin OR mitoxantrone OR 5-fluorouracil OR paclitaxel OR cyclophosphamide) (All Fields) | 724900 |
| #3 | (“three-dimensional speckle tracking” OR “three-dimensional spot tracking” OR echocardiography OR ultrasonography OR ultrasound) (All Fields) | 593184 |
| #4 | (deformation OR strain) (All Fields) | 1734295 |
| #5 | #1 AND #2 AND #3 AND #4 | 411 |
