# Supplementary material for: Valor Diagnóstico de Parâmetros Tridimensionais de Strain de Imagem de Speckle Tracking para Detecção de Disfunção Cardíaca Relacionada à Quimioterapia do Câncer: Uma Metanálise
Source: Arq Bras Cardiol. 2023 Jul 25;120(8):e20220370. [Article in Portuguese] doi: 10.36660/abc.20220370 (PMC10464855; doi:10.36660/abc.20220370)
Supplement: Supplementary file 2 [file 2022-0370_AO_SupplementaryTable2.pdf]

Table S2. Information (Vendors, Echocardiographic parameters, Data analysis) about 3D-STI.

Study	Vendors	Echocardiographic parameters	Data analysis
Chen, J 2019	A GE Vivid E9 color Doppler ultrasound system (GE Healthcare) and an M5S single crystal probe (4V-D) with a probe frequency of 1.7-3.3 MHz were used.	The system automatically calculates the parameter values.	The image was imported into the workstation running software (Echopac PC, 110.1.1, GE Healthcare), and data analysis was performed with the Echo Pac-110 analysis software.
Coutinho Cruz, M 2020	Vivid 95 or Vivid 9 ultrasound systems (GE Healthcare) were used to acquire parasternal long and short-axis views, as well as apical 4-, 2-, and 3-chamber views.	Echocardiographic parameters were determined according to the American Society of Echocardiography/European Association of Cardiovascular Imaging's recommendations.	Data sets were analyzed on a workstation (EchoPAC BT12 workstation, GE Healthcare).
Guan, J 2021	A GE Vivid E95 or GE Vivid E9 color Doppler ultrasound with an M5S-D probe (probe frequency 1.4-4.6 Hz) and a 4V-D probe (probe frequency 1.5-4.0 Hz).	The measurement of GLS and myocardial work was based on automated function imaging.	All strain analyses were performed using semiautomatic speckle tracking technology (EchoPAC203, GE Medical System, Milwaukee, Wisconsin) using the entire left ventricular model (the three apical views).
Mihalcea, D 2020	All ultrasound examinations were performed with a commercially available system equipped with a 4 V probe for 3DE (Vivid E9 Dimension, GE Medical Systems, Horten, Norway).	Deformation parameters were automatically calculated for each of 17 LV segments after tracking was confirmed visually.	Digital achieved data were analyzed offline using a dedicated software package (EchoPac version BT 12 for PC; GE Medical Systems) with 4D auto-LVQ system.
Mornos, C 2014	A commercially available 3D matrix array transducer (Vivid E9 scanner, 3V-D probe, 2.5 MHz, GE Vingmed Ultrasound, Horten, Norway).	NR	Data analysis was performed using the original raw data from all 3D echocardiographic data sets on an EchoPAC software workstation (version BT11, 4D Auto LVQ; GE Healthcare, UK).

Song, FY 2017	A commercially available ultrasound machine (iE33, Philips Medical Systems, Andover WA, USA) equipped with S5-1 (1-5 MHz) and X3-1 (1-3 MHz).	Echocardiographic parameters were determined according to the American Society of Echocardiography.	TomTec 4D LV analysis (4.6.0.411, TomTec Imaging Systems GMBH, Germany) was performed for 3D-STE data analysis.
Wang, B 2020	An ultrasound instrument (iE33; Philips Medical Systems) equipped with S5-1 and X3, a 3D volumetric transducer.	Global longitudinal strain (GLS) value was averaged of 17 ventricular segments from the three standard apical views.	Speckle tracking analyses were conducted offline with dedicated software (QLAB version 8.1; Philips Medical System).
Wang, Z 2021	GE Vivid E9-type echocardiography instrument (USA), equipped with three dimensional (4V) probes with EchoPAC workstation. Probe frequency range of about 1.7-3.3 MHz, with a scanning depth of 14-22 cm.	NR	Dynamic images of the four-chamber apical views were imported into the EchoPAC workstation.
Zhai, Z 2021	The 4 V, 1.5 to 4.0 MHz probe of a Vivid E9 ultrasound system (GE-Vingmed, Horten, Norway) was used.	Deformation parameters were automatically calculated for each of 17 LV segments after tracking was confirmed visually.	Data stored in a EchoPAC 7.0 workstation (GE-Vingmed, Horten, Norway). The 3D-STI analysis software was used for post-processing.
