# Supplementary material for: Valor Diagnóstico de Parâmetros Tridimensionais de Strain de Imagem de Speckle Tracking para Detecção de Disfunção Cardíaca Relacionada à Quimioterapia do Câncer: Uma Metanálise
Source: Arq Bras Cardiol. 2023 Jul 25;120(8):e20220370. [Article in Portuguese] doi: 10.36660/abc.20220370 (PMC10464855; doi:10.36660/abc.20220370)
Supplement: Supplementary file 3 [file 2022-0370_AO_Figure_S1.pdf]

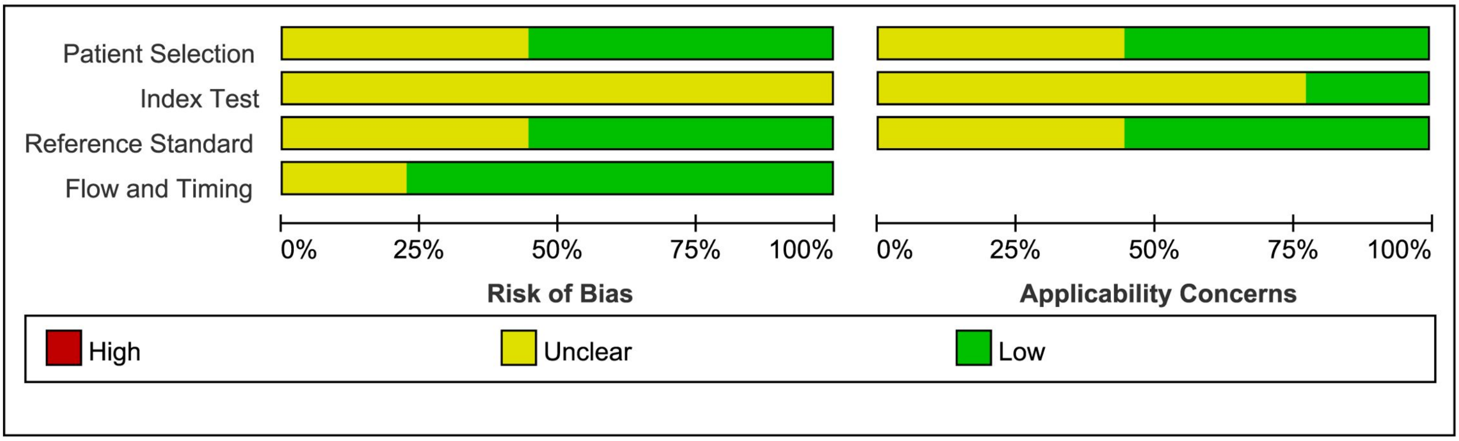
Patient Selection
Index Test
Reference Standard
Flow and Timing
0%
25%
50%
75%
100%
Risk of Bias
Applicability Concerns
High
Unclear
Low

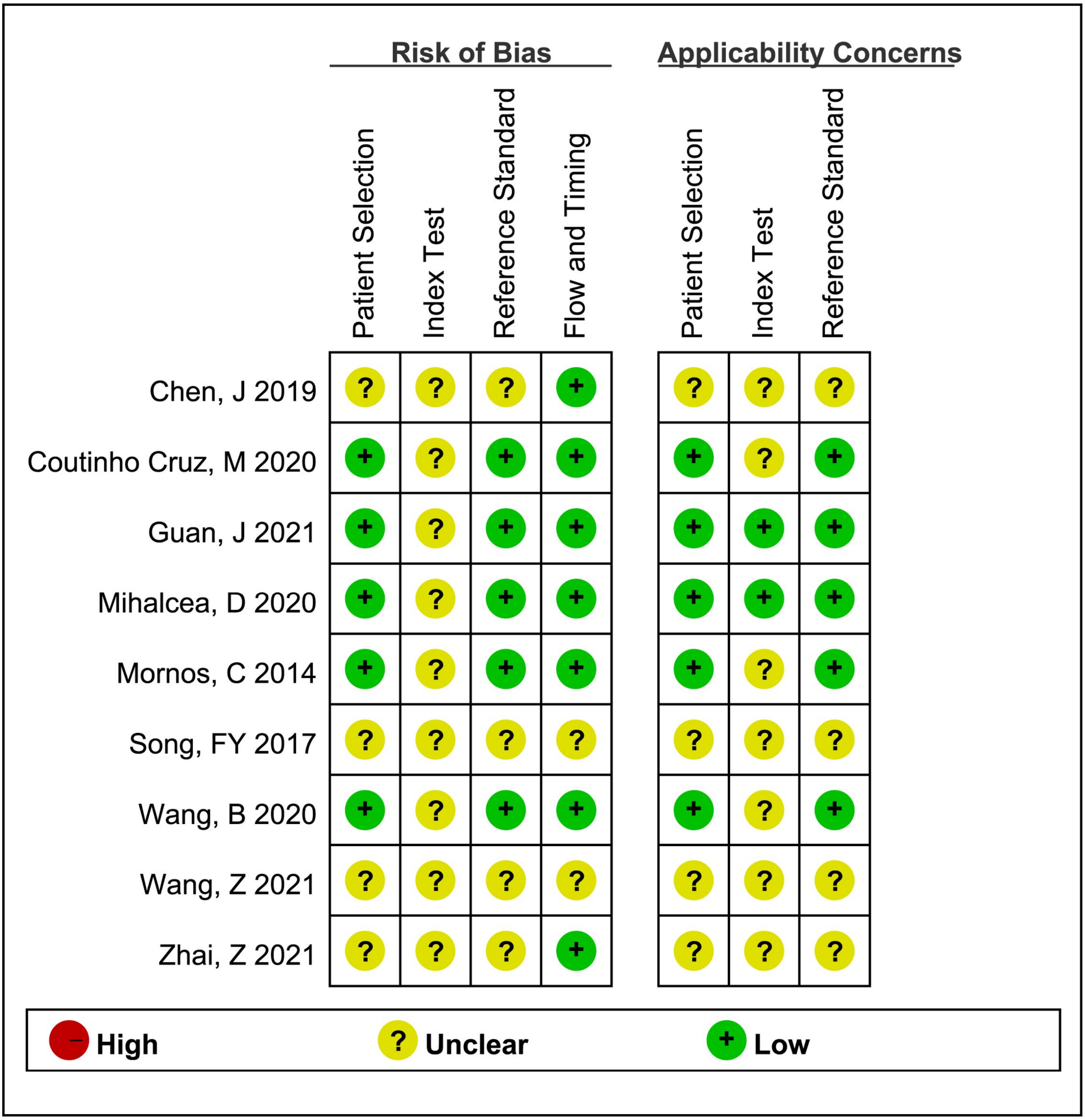
Risk of Bias
Applicability Concerns
Patient Selection
Index Test
Reference Standard
Flow and Timing
Chen, J 2019
Coutinho Cruz, M 2020
Guan, J 2021
Mihalcea, D 2020
Mornos, C 2014
Song, FY 2017
Wang, B 2020
Wang, Z 2021
Zhai, Z 2021
High
Unclear
Low
