# Supplementary material for: Valor Diagnóstico de Parâmetros Tridimensionais de Strain de Imagem de Speckle Tracking para Detecção de Disfunção Cardíaca Relacionada à Quimioterapia do Câncer: Uma Metanálise
Source: Arq Bras Cardiol. 2023 Jul 25;120(8):e20220370. [Article in Portuguese] doi: 10.36660/abc.20220370 (PMC10464855; doi:10.36660/abc.20220370)
Supplement: Translation

# Translation: Diagnostic Value of Three-Dimensional Speckle Tracking Imaging Strain Parameters for Detection of Cancer Chemotherapy-Related Cardiac Dysfunction: A Meta-Analysis

**Authors:** Yingying Guan

**Affiliations:** 1 China Taizhou Central Hospital (Taizhou University Hospital), Taizhou – China

**Keywords:** Anthracyclines, Cardiotoxicity, Heart Failure, Drug Therapy, Neoplasms

## Abstract

**Background:**

Chemotherapeutic agents (e.g., anthracyclines, trastuzumab) commonly used for treating malignant tumors have been demonstrated to have cardiotoxic effects, which is associated with poor prognosis. Three-dimensional echocardiography has been used to predict cancer chemotherapy-induced cardiac dysfunction.

**Objectives:**

Evaluation of the diagnostic performance of strain parameters, global area strain (GAS), longitudinal strain (GLS), circumferential strain (GCS), and radial strain (GRS) by meta-analysis.

**Methods:**

Relevant studies were searched from the Embase, PubMed, and Web of Science databases. Statistical analysis was performed using Stata 12. The summary receiver operating characteristic curve, sensitivity, specificity, positive likelihood ratio (PLR), negative likelihood ratio (NLR), and corresponding 95% confidence interval for the four strain parameters were pooled. P<0.05 was considered statistically significant.

**Results:**

Nine studies involving 650 participants were included. GAS and GLS showed significant diagnostic advantages over GCS and GRS. For GAS, the sensitivity was 0.85 (0.70, 0.93) and specificity was 0.82(0.78, 0.86) with PLR of 4.76 (3.55, 6.39) and NLR of 0.18 (0.09, 0.39) and an area under the curve (AUC) of 0.85 (0.82, 0.88). For GLS, the sensitivity was 0.81 (0.74, 0.86) and specificity was 0.81(0.68, 0.90) with PLR of 4.35(2.42, 7.80) and NLR of 0.23 (0.17, 0.33) and an AUC of 0.85 (0.82, 0.88). The GCS showed a sensitivity of 0.63 and a specificity of 0.79 with an AUC of 0.77. The GRS showed a sensitivity of 0.74 and a specificity of 0.66 with an AUC of 0.73.

**Conclusion:**

3D-STI strain parameters GAS and GLS showed good performance in detecting early cardiac dysfunction in patients with tumors receiving chemotherapy.

## Introduction

The prognosis of patients with tumors has greatly improved with the development of tumor therapy methods, while complications related to tumor treatment drugs have become increasingly prominent, which has been an important problem affecting patient survival.^1^ Chemotherapy remains the primary treatment method for various tumors. However, while killing cancer cells, chemotherapeutic drugs also cause damage to many normal tissues and cells throughout the body, among which the damage of myocardial cells is a common concurrent change during chemotherapy.^2 - 4^ For example, anthracyclines, a class of highly effective broad-spectrum anticancer drugs that can improve patient survival and reduce tumor recurrence and metastasis, has been reported to cause dose-related cardiotoxicity, resulting in irreversible cardiac damage, which negatively affects prognosis.^5 , 6^ The overall incidence of cardiotoxicity (left ventricular ejection fraction [LVEF] decrease > 10% from baseline and a final value < 50 %) of 9% was observed in a heterogeneous cohort of 2625 patients with cancer who received anthracycline-containing treatment in a median follow-up duration of 5.2 years.^7^ A previous study reported a 16%, 32%, and 65% rate of left ventricular dysfunction (ejection fraction decrease > 10% below normal) under cumulative doses of 300, 400, and 550 mg/m^2^ , respectively.^8^ Trastuzumab, a targeted therapy for HER-2 overexpressing tumours, has greatly succeeded in breast cancer.^9^ However, trastuzumab was reported to be associated with loss of LV contractile function and heart failure.^10^ Therefore, early detection and active prevention of cardiac dysfunction caused by cancer chemotherapeutic agents are necessary.

Transthoracic echocardiography has been widely recommended for monitoring cardiac function in patients receiving chemotherapy.^11^ Traditional indicators of LVEF have several limitations; particularly, they are insensitive in determining early, subtle changes in myocardial function.^12^ Three-dimensional speckle tracking imaging (3D-STI) is a newly developed ultrasonographic examination method that can trace the motion track of the acoustic speckle of the myocardium in the region of interest in the 3D space and describe the deformation degree of the myocardial tissue to depict the curve of cardiac function change.^13 , 14^ Compared with 2D-STI, 3D-STI was more accurate in identifying endocardium boundary, and showed superiority for evaluating abnormal motion of local ventricular wall and quantifying LV mass.^15^ In addition, 3D-STI was more feasible and reproducible for LV function assessment, taking less time than 2D-STI.^16^ 3D-STI is a noninvasive and highly effective method in assessing cardiac function and has been applied in diagnosing subclinical myocardial dysfunction caused by various pathogenes.^17 - 19^ 3D-STI involves multiparameter analysis, including global area strain (GAS), longitudinal strain (GLS), radial strain (GRS), and circumferential strain (GCS). GLS is the best-studied strain parameter and is demonstrated to show both diagnostic and prognostic value; GLS changes have been considered an early marker of cardiotoxicity.^20^ Mornoş et al. indicated that GLS had independent predictive value for cardiotoxicity.^21^ While studies have suggested that GAS is more feasible than conventional strain parameters in detecting early LV systolic dysfunction, it is a sensitive and repeatable parameter.^22 , 23^ For breast cancer patients who received six cycles of epirubicin+cyclophosphamide chemotherapy, GAS, GLS, and GCS markedly decreased during mid- and end-chemotherapy, while there was no obvious changes in GRS after chemotherapy; in addition, GAS showed greatest area under the curve (AUC) than other strain parameters.^24^ In study of Galderisi et al., GAS, GLS and GRS markedly changed in characterizing early LV structure and function abnormalities, while GCS showed no changes.^25^ These studies showed inconsistent views on the clinical value of these strain parameters in determining cardiac dysfunction.

This meta-analysis aims to assess the overall diagnostic advantages of the four 3D-STI strain parameters in determining cardiac function injury in patients with cancer after chemotherapy.

## Methods

### Study Collection

From the Embase, PubMed, and Web of Science databases, relevant studies were systematically collected based on the pre-established search strategy with a retrieval time of up to March 11, 2022. The search terms included four categories: 1) “dysfunction” OR “heart failure” OR “cardiotoxicity” OR “cardiotox”; 2) “chemotherapy” OR “doxorubicin” OR “daunorubicin” OR “trastuzumab” OR “epirubicin” OR “idarubicin” OR “mitoxantrone” OR “anthracycline” OR “cyclophosphamide” OR “Adriamycin” OR “paclitaxel” OR “5-fluorouracil”; 3) “three-dimensional speckle tracking” OR “three-dimensional spot tracking” OR echocardiography OR ultrasonography OR ultrasound; and 4) deformation OR strain. The four categories of search terms were combined with “AND.” The search strategies for the different databases are presented in detail in Table S1. Moreover, relevant studies in the paper version were also manually collected. The cited references in the included studies and relevant reviews were also obtained.

### Study selection

Studies were included if they met the following criteria: 1) studies that included patients with cancer who received chemotherapy for the first time; 2) studies that recorded the diagnostic value of each strain parameter of 3D-STI in cardiac function injury, including GAS, GCS, GLS, and GRS; and 3) studies that provided data in terms of the diagnostic performance of each strain parameter of 3D-STI in cardiac function injury, including true positive (TP), true negative (TN), false positive (FP), and false negative (FN). Studies that met the following criteria were excluded from this meta-analysis: 1) conference abstracts, comments, reviews, and other non-original articles; 2) studies that included patients who had a history of heart disease or patients who previously received drugs or radiotherapy that cause cardiotoxicity; and 3) multiple studies that reported the data from the same participate populations, only the study with the most complete data for analysis was included.

### Data extraction and quality evaluation

Data were extracted from the included studies by two independent investigators (YY-Guan and JY-Zhou), and any unconformity was settled through consultation. The extracted data included the following: year of publication, the first author’s name, region of research, and information on the participants involved in the studies, including sample size, age, sex, TP, FP, TN, FN, cancer types, and chemotherapy regimens. The definition of cardiac dysfunction and the vendors for the ultrasound system were also extracted. QUADAS 2^26^ was used to assess the methodological quality of the studies.

### Statistical analysis

Statistical analysis was performed using the MIDAS command (bivariate mixed-effect model) provided in Stata 12 (version 12 SE). The sensitivity, specificity, positive and negative likelihood ratios (PLR and NLR, respectively), summary receiver operating characteristic (SROC) curve, and corresponding 95% confidence interval (CI) were reported. The I^2^ test and Cochran’s Q test were used to assess the heterogeneity of studies,^27^ and significant heterogeneity was detected when the Q statistic p-value was < 0.05 and/or I^2^ > 50%. Meta-regression was conducted to investigate the influences of regions, the definition of cardiac dysfunction, vendors, and the type of cancer on the pooled results. Publication bias among the studies was assessed using Deek’s funnel plot.^28^ Spearman correlation analysis was used to evaluate threshold effects. A p-value < 0.05 indicated the presence of a significant threshold effect.^29^

## Results

### Study retrieval

From the Embase, PubMed, and Web of Science databases, 1032, 447, and 411 studies were collected, respectively. Of these, 655 duplicate studies were excluded; 1213 irrelevant studies were removed by reading the title/abstract. Among the remaining 22 studies, six studies involving 2D-STIs, four without available data, two reviews, and one study involving duplicated participants were excluded by full-text reading. As a result, nine studies^21 , 24 , 30 - 36^ that met the predesigned inclusion and exclusion criteria were selected for this meta-analysis ( Figure 1 ).

**Figure 1:** – Flowchart of the study selection process.

### Characteristics of the included studies

All nine included studies were prospective cohort studies published between 2014 and 2021, and a total of 650 participants were included, with 185 men and 465 women. Table 1 presents detailed information on the nine included studies. These studies have been conducted in China, Portugal, Romania, and Greece. Among the nine included studies, patients with breast cancer were analyzed in three studies,^24 , 30 , 31^ patients with diffuse large B-cell non-Hodgkin’s lymphoma were examined in two studies,^33 , 34^ and patients with colorectal cancer,^35^ non-Hodgkin’s lymphoma^32^ and ovarian cancer^36^ were included in one study. In a study by Mornos et al.,^21^ multiple cancer types were involved, including acute lymphoblastic leukemia, acute myeloblastic leukemia, breast cancer, non-Hodgkin’s lymphoma, Hodgkin’s lymphoma, and osteosarcoma. Patients were confirmed histopathologically or cytopathologically in four studies,^24 , 31 , 33 , 34^ and no available data were reported in the other five studies.^21 , 30 , 32 , 35 , 36^ A decrease in LVEF was used as a criterion for determining impaired cardiac function in five studies,^21 , 30 - 32 , 34^ and the claim that cardiotoxicity occurred in patients with cancer after chemotherapy was suggested in the remaining four studies.^24 , 33 , 35 , 36^ In addition to the Philips ultrasound system used in the study of Song et al.^33^ and Wang et al.,^34^ a Vivid E9 color Doppler ultrasound system (GE Healthcare) was used in the other studies, as shown in Table S2 . The cut-off values of TP, FP, TN, and FN for GAS, GLS, GCS, and GRS parameters of 3D-STI in diagnosing cardiac function injury are shown in Table 2 . The methodological quality evaluation showed that the bias of these studies was moderate, indicating a moderate methodological quality ( Figure S1 ).

**Table 1:** – Characteristics of 9 included studies in this meta-analysis

Study	Area	Type of cancer	Confirmed of cancer	Chemotherapy regimen	n, M/F	Age, years	CD (%)	Definition of CD
Chen, J 2019	China	Breast cancer	Pathologically	EC	83, 0/83	49.25±8.75	25.0%	LVSD after receiving chemotherapy
Coutinho Cruz, M 2020	Portugal	Breast cancer	NR	Anthracyclines	105, 0/105	53.8±12.5	21.0%	Absolute decrease in LVEF > 10% to a value < 54%
Guan, J 2021	China	Breast cancer	Histology or cytopathology	G1: T+Cb, G2:A+E+C+T, G3: H+G2	79, 0/79	48 (22-66)	11.4%	Reduction of LVEF by ≥5% to <53% with symptoms of heart failure or an asymptomatic reduction of LVEF by ≥10% to <53%
Mihalcea, D 2020	Romania	NHL	NR	CHOP	110, 51/59	58±11	15.6%	LVEF reduction below 50%, with more than 10 percentage points
Mornos, C 2014	Greece	Breast, NHL, HL, ALL, AML, Osteosarcoma	NR	Anthracyclines	59, 24/35	51±10	13.6%	Reduction of LVEF by ≥5% to <55% with symptoms of heart failure or an asymptomatic reduction of LVEF by ≥10% to <55%
Song, FY 2017	China	DLBCL	Histopathologically	R-CHOP	89, 60/29	49.3±12.5	50.0%	Subclinical systolic dysfunction after receiving chemotherapy
Wang, B 2020	China	DLBCL	Histopathologically	(R)-CHOP	65, 31/34	51.3±13.5	12.3%	Reduction in LVEF greater than 10%, to a value<53%, confirmed by a repeat echocardiography
Wang, Z 2021	China	CRC	NR	Carboplatin+	30, 19/11	50.2±7.45	75.0%	The latent toxicity in the left ventricular myocardium after receiving chemotherapy
Zhai, Z 2021	China	Ovarian cancer	NR	Paclitaxel	30, 0/30	51.6±7.4	50.0%	LVSD after receiving chemotherapy

*ALL: Acute lymphoblastic leukemia; AML: Acute myeloblastic leukemia; HL: Hodgkin’s lymphoma; T: Docetaxel; Cb: Carboplatin; A: Doxorubicin; E: Epirubicin; C: Cyclophosphamide; H: Trastuzumab; NHL: non-Hodgkin’s lymphoma; CRC: colorectal cancer; EC: epirubicin combined cyclophosphamide; (R)-CHOP: (rituximab)+cyclophosphamide+doxorubicin+vincristine+prednisone; mFOLFOX6: oxaliplatin+5-fluorouracil+calcium leucovorin; DLBCL: diffuse large B cell non-Hodgkin lymphoma; F: female; M: male; NR: not reported; CD: cardiac dysfunction; LVSD: left ventricular systolic dysfunction; LVEF: left ventricular ejection fraction. P<0.05 was considered statistically significant in all the included studies.*

**Table 2:** – TP/FN/FP/TN of 3D-STI parameters

Study	Country	Cut-off	True positive	False negative	False positive	True negative
**GAS**						
Chen, J 2019	China	-31.5%	68	15	49	200
Mihalcea, D 2020	Romania	-28.0%	15	2	16	76
Wang, Z 2021	China	-28.0%	64	26	4	26
Zhai, Z 2021	China	-32.0%	29	1	3	26
**GLS**						
Chen, J 2019	China	-16.5%	62	21	116	133
Guan, J 2021	China	-22.7%	5	4	4	66
Mihalcea, D 2020	Romania	-19.0%	16	2	14	78
Mornos, C 2014	Greece	-13.7%	7	1	15	36
Song, FY 2017	China	-20.4%	71	18	27	62
Wang, B 2020	China	-13.8%	6	2	5	49
Wang, Z 2021	China	-20.0%	80	10	11	19
Zhai, Z 2021	China	-17.0%	25	5	1	29
**GCS**						
Chen, J 2019	China	-17.5%	57	26	116	133
Coutinho Cruz, M 2020	Portugal	-34.2%	14	8	20	55
Guan, J 2021	China	-16.5%	6	3	13	57
Mihalcea, D 2020	Romania	-37.0%	14	4	17	74
Song, FY 2017	China	-29.2%	62	27	36	53
Wang, Z 2021	China	-18.0%	19	71	1	29
Zhai, Z 2021	China	-19.0%	25	5	3	27
**GRS**						
Chen, J 2019	China	44.5%	50	33	103	146
Coutinho Cruz, M 2020	Portugal	34.4%	16	6	25	55
Mihalcea, D 2020	Romania	43.0%	11	7	28	64
Wang, Z 2021	China	45.0%	67	23	15	15
Zhai, Z 2021	China	50.0%	27	3	7	23

*GAS: global area strain; GLS: longitudinal strain; GCS: circumferential strain; GRS: radial strain.*

### Diagnostic value of GAS

The diagnostic value of GAS in 3D-STI for cardiac function injury in patients with cancer has been reported in four studies. Spearman correlation analysis revealed no significant threshold effect (p = 1.00). The pooled sensitivity was 0.85 (0.70, 0.93) with significant heterogeneity. The pooled specificity was 0.82 (0.78, 0.86), and no significant heterogeneity was observed ( Figure 2A ). The pooled PLR was 4.76 (3.55, 6.39) without significant heterogeneity, while the pooled NLR was 0.18 (0.09, 0.39) with significant heterogeneity ( Figure 2B ). The SROC curve showed an area under the curve (AUC) of 0.85 (0.82, 0.88), suggesting the good diagnostic performance of GAS in predicting cardiac function injury in patients who underwent chemotherapy ( Figure 2C ). There was no significant publication bias among the four studies ( Figure 2D ).

**Figure 2:** – Pooled results showing the diagnostic value of GAS. Forest plots showing the pooled sensitivity and specificity (A) and PLR and NLR (B) of GAS in diagnosing cardiac function injury in patients receiving chemotherapy. SROC curves show the diagnostic performance of GAS (C). Deek’s funnel plot asymmetry test shows publication bias among studies (D). PLR: positive likelihood ratio; NLR: negative likelihood ratio; SROC: summary receiver operating characteristic; GAS: global area strain.

### Diagnostic value of GLS

The diagnostic value of GLS in 3D-STI for cardiac function injury in patients with cancer was reported in eight studies. The Spearman correlation analysis detected No significant threshold effect (p = 0.05). The pooled sensitivity and specificity were 0.81 (0.74, 0.86) and 0.81 (0.68, 0.90), and significant heterogeneity was found among these studies ( Figure 3A ). The pooled PLR and NLR were 4.35 (2.42, 7.80) and 0.23 (0.17, 0.33), respectively, and significant heterogeneity among studies was observed ( Figure 3B ). The SROC curve showed an AUC of 0.85 (0.82, 0.88), suggesting the good diagnostic performance of GLS in predicting cardiac function injury in patients who underwent chemotherapy ( Figure 3C ). No significant publication bias was observed ( Figure 3D ).

**Figure 3:** – Pooled results showing the diagnostic value of GLS.Forest plots showing the pooled sensitivity and specificity (A) and PLR and NLR (B) of GAS in diagnosing cardiac function injury in patients receiving chemotherapy. SROC curves show the diagnostic performance of GAS (C). Deek’s funnel plot asymmetry test shows publication bias among studies (D). PLR: positive likelihood ratio; NLR: negative likelihood ratio; SROC: summary receiver operating characteristic; GLS: global longitudinal strain.

### Diagnostic value of GCS

Seven studies reported the diagnostic value of GCS in 3D-STI for cardiac function injury in patients with cancer. The Spearman correlation analysis found No significant threshold effect (p=0.39). Significant heterogeneity was observed among the studies in terms of sensitivity and specificity. The pooled sensitivity and specificity were 0.63 (0.47, 0.77) and 0.79 (0.64, 0.89), respectively ( Figure 4A ). The pooled PLR and NLR were 2.99 (1.81, 4.93) and 0.46 (0.32, 0.68), respectively, and significant heterogeneity among studies was observed ( Figure 4B ). The SROC curve indicated an AUC of 0.77 (0.74, 0.81) ( Figure 4C ). No significant publication bias among the studies was detected using Deek’s funnel plot ( Figure 4D ).

**Figure 4:** – Pooled results showing the diagnostic value of GCS. Forest plots showing the pooled sensitivity and specificity (A) and PLR and NLR (B) of GAS in diagnosing cardiac function injury in patients receiving chemotherapy. SROC curves show the diagnostic performance of GAS (C). Deek’s funnel plot asymmetry test shows publication bias among studies (D). PLR: positive likelihood ratio; NLR: negative likelihood ratio; SROC: summary receiver operating characteristic; GCS: global circumferential strain.

### Diagnostic value of GRS

Five studies reported the diagnostic value of the GRS in 3D-STI for cardiac function injury in patients with cancer. Spearman correlation analysis indicated no significant threshold effect (p=1.00). The pooled sensitivity and specificity were 0.74 (0.63, 0.82) and 0.66 (0.59, 0.72), respectively, and significant heterogeneity was observed among studies ( Figure 5A ). A significant heterogeneity for PLR and NLR was observed among the studies. The pooled PLR and NLR values were 2.15 (1.62, 2.86) and 0.40 (0.26, 0.61), respectively ( Figure 5B ). The SROC curve showed an AUC of 0.73 (0.69, 0.77) ( Figure 5C ). No significant publication bias was observed among the studies ( Figure 5D ).

**Figure 5:** – Pooled results showing the diagnostic value of GRS. Forest plots showing the pooled sensitivity and specificity (A) and PLR and NLR (B) of GAS in diagnosing cardiac function injury in patients receiving chemotherapy. SROC curves show the diagnostic performance of GAS (C). Deek’s funnel plot asymmetry test shows publication bias among studies (D). PLR: positive likelihood ratio; NLR: negative likelihood ratio; SROC: summary receiver operating characteristic; GRS: global radial strain.

### Meta-regression

Meta-regression was conducted to investigate the influences of regions, the definition of cardiac dysfunction, vendors, and the type of cancers on the pooled sensitivity and specificity ( Table 3 ). Meta-regression was not performed for GAS because only four studies reported the diagnostic value of GAS for predicting cardiac dysfunction. For GCS, all these variables had no significant influences on pooled sensitivity and specificity. For GLS, the definition of cardiac dysfunction and type of cancers significantly influenced pooled sensitivity. For GRS, regions showed significant influences on pooled specificity, and the influences of type of cancers on pooled sensitivity and specificity were statistically significant.

**Table 3:** – Results of meta-regression

Factors	Category	No. of studies	Sensitivity	p value	Specificity	p value
**GLS**						
Regions	China	6	0.80 (0.73, 0.87)	0.1	0.82 (0.70, 0.94)	0.93
	Europe	2	0.89 (0.76, 1.00)		0.79 (0.56, 1.00)	
Definition of CD	Reduction of LVEF	4	0.79 (0.66, 0.92)	0.04	0.87 (0.78, 0.96)	0.77
	Chemotherapy toxicity	4	0.82 (0.76, 0.88)		0.72 (0.56, 0.89)	
Vendors	GE	6	0.82 (0.75, 0.89)	0.1	0.81 (0.68, 0.94)	0.68
	Philips	2	0.79 (0.68, 0.90)		0.82 (0.62, 1.00)	
Type of cancers	Breast	2	0.69 (0.55, 0.83)	<0.01	0.80 (0.58, 1.00)	0.64
	Non-Breast	6	0.83 (0.78, 0.89)		0.82 (0.69, 0.94)	
**GCS**						
Regions	China	5	0.60 (0.42, 0.79)	0.45	0.79 (0.64, 0.94)	0.78
	Europe	2	0.71 (0.44, 0.98)		0.78 (0.56, 1.00)	
Definition of CD	Reduction of LVEF	3	0.70 (0.47, 0.93)	0.77	0.79 (0.62, 0.96)	0.69
	Chemotherapy toxicity	4	0.60 (0.40, 0.79)		0.78 (0.61, 0.95)	
Vendors	GE	6	0.62 (0.45, 0.80)	0.69	0.81 (0.70, 0.92)	0.31
	Philips	1	0.70 (0.35, 1.00)		0.60 (0.21, 0.98)	
Type of cancers	Breast	3	0.65 (0.41, 0.89)	0.96	0.70 (0.50, 0.90)	0.11
	Non-Breast	4	0.61 (0.41, 0.82)		0.84 (0.72, 0.97)	
**GRS**						
Regions	China	3	0.75 (0.62, 0.87)	0.83	0.62 (0.54, 0.70)	0.01
	Europe	2	0.68 (0.48, 0.88)		0.69 (0.61, 0.77)	
Definition of CD	Reduction of LVEF	2	0.68 (0.48, 0.88)	0.25	0.69 (0.61, 0.77)	0.28
	Chemotherapy toxicity	3	0.75 (0.62, 0.87)		0.62 (0.54, 0.70)	
Type of cancers	Breast	2	0.65 (0.53, 0.77)	0.01	0.63 (0.54, 0.71)	0.04
	Non-Breast	3	0.77 (0.69,0.86)		0.67 (0.58, 0.76)	

*GAS: global area strain; GLS: longitudinal strain; GCS: circumferential strain; GRS: radial strain; CD: cardiac dysfunction; LVEF: left ventricular ejection fraction*

## Discussion

### Main Findings

This meta-analysis revealed that the 3D-STI strain parameters GAS and GLS showed high sensitivity (0.85 for GAS, 0.81 for GLS) and specificity (0.82 for GAS, 0.81 for GLS) in the diagnosis of cardiac function injury in patients with cancer after chemotherapy, with an AUC of 0.85 for both strain parameters, suggesting good diagnostic performance. GCS and GRS showed relatively poorer diagnostic advantages in determining cardiac function injury in patients with cancer after chemotherapy, with a sensitivity of 0.63 and specificity of 0.79 with an AUC of 0.77 for GCS and sensitivity of 0.74 and specificity of 0.66 with an AUC of 0.73 for GRS ( Central Illustration ).

**Central Illustration:** : Diagnostic Value of Three-Dimensional Speckle Tracking Imaging Strain Parameters for Detection of Cancer Chemotherapy-Related Cardiac Dysfunction: A Meta-Analysis

*Central illustration of the results of the article. Four 3D-STI strain parameters in predicting cancer chemotherapy-related cardiac dysfunction were evaluated by meta-analysis. GAS showed the best diagnostic performance.*

### Diagnostic performance of each parameter in previous studies

Previous studies have demonstrated that GAS is the most sensitive strain parameter reflecting left ventricular myocardial function changes.^23 , 37^ For patients showing risk factors for the development of heart failure, the GAS displayed a sensitivity of 86.3% and specificity of 88.4% in determining early left ventricular systolic dysfunction.^23^ Piveta et al. indicated that, in patients with breast cancer who received a lower cumulative dose of doxorubicin (120 mg/m^2^ ), only the 3D-STI strain parameter GAS changed, and its change was correlated with decreased LVEF (definitive cardiotoxicity).^38^ GAS is an index introduced late, representing the percentage of deformation in the LV endocardial surface area. The diagnostic advantages of GAS are probably achieved by a more comprehensive evaluation by integrating both longitudinal and circumferential motion of the myocardium.^23^ In addition, the endocardium is the most prone to ischemia, which reasonably explains the good performance of GAS in detecting changes in left ventricular function.^23^ Oikonomou et al. indicated that GLS had a good predictive performance for cancer therapy-related cardiac dysfunction with an odds ratio of 12.27 and AUC of 0.86.^39^ Reduction in GLS was predictive of decreased LVEF in patients receiving anthracyclines. For patients with tumors with normal LVEF, an impaired GLS ≥ -18% showed good predictive value for increased risk of anthracycline-induced cardiac dysfunction (defined as a decrease in ejection fraction > 10% and final value < 50%) and increased cardiovascular-related mortality.^40^ For patients with tumors receiving a cumulative anthracycline dose of 150 mg/m^2^ , the obtained GLS (threshold of -17.45%) showed the strongest predictive value for cardiotoxicity, with an AUC of 0.82, and might be used as an independent predictive factor.^41^ During the evaluation of the functional variation of the left ventricular segmental systole for patients with lymphoma receiving anthracycline chemotherapy, a significant reduction in 3D-GLS from baseline was observed following four cycles of anthracycline treatment, while no variation in 3D-GCS was observed.^19^ Chen et al. evaluated early myocardial damage in patients with breast cancer receiving anthracycline chemotherapy using 3D-STI, and no variation in GRS was observed after anthracycline therapy.^24^ To assess the functional variation in the right ventricular myocardium in patients with breast cancer using 3D-STI, right ventricular GAS, and GLA were markedly decreased, while GCS and GRS were unchanged after pirarubicin chemotherapy.^42^ Consistent with our meta-analysis results, these studies findings suggested that GAS and GLS had good predictive value, while GCS and GRC showed low diagnostic advantages in detecting cardiotoxicity in patients with tumors receiving chemotherapy. However, the superiority in the diagnostic performance of GAS is obtained based on only four studies and should be further confirmed.

### Causes of heterogeneity

There was significant heterogeneity among studies in reporting some outcome indices, and there were also differences in diagnostic criteria, chemotherapy regimen, cancer type, and criteria for heart function impairment among the participants in different studies. Meta-regression was conducted to investigate the potential sources of heterogeneity and found that definition of cardiac dysfunction (reduction of LVEF and chemotherapy toxicity), regions that the study conducted (China and Europe), and type of cancers (breast cancer and non-breast cancer) might be sources of heterogeneity, especially type of cancers. Type of cancers showed significant influences on GLS’s pooled sensitivity and GRS’s sensitivity and specificity. The 3D strain parameters showed poor agreement inter-vendors, and it was suggested to use the same 3D STE platform to obtain the baseline and follow-up data in longitudinal studies.^43^ Among the included studies in this meta-analysis, the VividE9 ultrasound system was used in most studies, while the Philips ultrasound system was also used in two studies. In meta-regression analysis, vendors showed no significant influences to sensitivity (p=0.1 for GLS, and p=0.69 for GCS) and specificity (p=0.68 for GLS, and p=0.31 for GCS), indicating that vendor was not a source of heterogeneity.

### Strengths and limitations

The diagnostic performance of the four 3D-STI strain parameters in detecting cardiac dysfunction in patients with tumors receiving chemotherapy was comprehensively evaluated in this meta-analysis. Among these, GAS and GLS showed good application prospects in this field. The methodological quality of the studies in the current meta-analysis was moderate, and no significant publication bias was detected in these studies, indicating the high reliability of the results. However, this study has some limitations. First, meta-regression was not conducted for GAS because only four studies reported the diagnostic value of GAS for predicting cardiac dysfunction. Second, although the AUC for GLS seems good, the cut-off values varied greatly among studies (from -13.7% to -22.7%). This was common in meta-analyses of diagnostic trials, probably attributed to the differences in diagnostic criteria, number of participants, chemotherapy regimen, cancer type, and criteria for heart function impairment among the participants in different studies. In addition, the meta-regression results indicated that the type of cancers significantly influenced pooled GLS sensitivity. Third, the number of included studies and involved participants was small, and more high-quality large-sample studies are required to verify the stability and extrapolation of the results.

## Conclusion

In conclusion, this meta-analysis indicated that the 3D-STI strain parameters GAS and GLS showed good performance in detecting cardiac dysfunction in patients with tumors receiving chemotherapy. However, the superiority in the diagnostic performance of GAS is obtained based on only four studies. Therefore, further investigations are needed to confirm these findings based on more high-quality large-sample studies. Clinically, in addition to GLS, more attention should be paid to the changes in GAS when evaluating cardiac dysfunction in patients with tumors receiving chemotherapy using 3D-STI.

## *Supplemental Materials

For supplementary table 1, please click here.



For supplementary table 1, please click here.



For supplementary figure, please click here.
